# Understanding the challenges of identifying, supporting, and signposting patients with alcohol use disorder in secondary care hospitals, post COVID-19: a qualitative analysis from the North East and North Cumbria, England

**DOI:** 10.1186/s12913-024-11232-4

**Published:** 2024-07-01

**Authors:** Katherine Jackson, Rosie Baker, Amy O’Donnell, Iain Loughran, William Hartrey, Sarah Hulse

**Affiliations:** 1 Newcastle University, Newcastle upon Tyne, UK; 2 North Tees and Hartlepool NHS Hospitals Foundation Trust, Stockton on Tees, UK; 3 North East Commissioning Service, Newcastle upon Tyne, UK; 4 Norfolk and Norwich University Hospitals NHS Foundation Trust, Norwich, UK; 5 North East and North Cumbria Integrated Care Board, Newcastle upon Tyne, UK

**Keywords:** Alcohol, Secondary care, Inequalities, Normalization process theory, Qualitative research

## Abstract

**Background:**

Alcohol-related mortality and morbidity increased during the COVID-19 pandemic in England, with people from lower-socioeconomic groups disproportionately affected. The North East and North Cumbria (NENC) region has high levels of deprivation and the highest rates of alcohol-related harm in England. Consequently, there is an urgent need for the implementation of evidence-based preventative approaches such as identifying people at risk of alcohol harm and providing them with appropriate support. Non-alcohol specialist secondary care clinicians could play a key role in delivering these interventions, but current implementation remains limited. In this study we aimed to explore current practices and challenges around identifying, supporting, and signposting patients with Alcohol Use Disorder (AUD) in secondary care hospitals in the NENC through the accounts of staff in the post COVID-19 context.

**Methods:**

Semi-structured qualitative interviews were conducted with 30 non-alcohol specialist staff (10 doctors, 20 nurses) in eight secondary care hospitals across the NENC between June and October 2021. Data were analysed inductively and deductively to identify key codes and themes, with Normalisation Process Theory (NPT) then used to structure the findings.

**Results:**

Findings were grouped using the NPT domains ‘implementation contexts’ and ‘implementation mechanisms’. The following implementation contexts were identified as key factors limiting the implementation of alcohol prevention work: poverty which has been exacerbated by COVID-19 and the prioritisation of acute presentations (negotiating capacity); structural stigma (strategic intentions); and relational stigma (reframing organisational logics). Implementation mechanisms identified as barriers were: workforce knowledge and skills (cognitive participation); the perception that other departments and roles were better placed to deliver this preventative work than their own (collective action); and the perceived futility and negative feedback cycle (reflexive monitoring).

**Conclusions:**

COVID-19, has generated additional challenges to identifying, supporting, and signposting patients with AUD in secondary care hospitals in the NENC. Our interpretation suggests that implementation contexts, in particular structural stigma and growing economic disparity, are the greatest barriers to implementation of evidence-based care in this area. Thus, while some implementation mechanisms can be addressed at a local policy and practice level via improved training and support, system-wide action is needed to enable sustained delivery of preventative alcohol work in these settings.

**Supplementary Information:**

The online version contains supplementary material available at 10.1186/s12913-024-11232-4.

## Background

Alcohol is now the leading risk factor for ill-health, early mortality, and disability amongst working age adults (aged 15 to 49) in England, and the fifth leading risk factor for ill-health across all age groups [1]. Evidence also shows significant socioeconomic inequalities in alcohol-related harm [2]. Over half of the one million hospital admissions relating to alcohol in England each year occur in the lowest three socioeconomic deciles [3] and rates of alcohol-related deaths increase with decreasing socioeconomic status [4]. In 2020 people under 75 years living in the most deprived areas in England had a 4.8 times greater likelihood of premature mortality from alcohol-related liver disease than those living in the most affluent areas [5].

Although globally, there is mixed evidence about the impact of the COVID-19 pandemic and associated social and economic restrictions on alcohol consumption [6], some studies suggest that people who were already drinking alcohol heavily increased their intake during this period [7, 8]. Latest data for England show that the total number of deaths from conditions that were wholly attributed to alcohol rose by 20% in a single year in 2020, the largest increase on record [9]. In England, and elsewhere, it has been argued that COVID-19 should be regarded as a syndemic rather than a pandemic, as it has interacted with, and most adversely affected those in the most deprived social groups who were already experiencing the greatest inequalities [10]. In the case of alcohol use, COVID-19 may have interacted with and exacerbated the social conditions associated with alcohol use such as poverty, and loneliness and isolation [11, 12]. Moreover, with evidence that alcohol-related harms will continue to increase, there is concern this will further widen health inequalities for those communities and regions who are likely to be most affected [8, 13]. Thus, there is an urgent need for the implementation of evidence-based preventative strategies to reduce alcohol harm and associated inequalities, as part of a wider system level approach that includes primary, secondary and specialist care settings [8]. From here we use the term Alcohol Use Disorder (AUD), to refer to a spectrum of alcohol use from harmful to dependent alcohol use [14].

In secondary care hospitals, the UK government prioritised the implementation of Alcohol Care Teams (ACTs) in England in the National Health Service (NHS) Long Term Plan with the aim of improving care and reducing alcohol-related harms [15]. ACTs are clinician-led, multidisciplinary teams designed to support provision of integrated alcohol treatment pathways across primary, secondary and community care, and have been shown to reduce alcohol harms through reductions in avoidable bed days; readmissions; Accident and Emergency Department (AED) attendances; and ambulance call outs [16]. However, the non-specialist secondary care workforce also has an essential role in identifying and managing people at risk, using evidence-based approaches such as screening patients for excessive alcohol use and the provision brief advice [17]. Given that people may not always present primarily with alcohol-related concerns, routine screening provides an important opportunity to identify people at an earlier stage in their drinking and thereby prevent escalation of alcohol-related problems. Current NHS clinical guidance [18] requires that non-specialist healthcare staff *‘should be competent to identify harmful drinking (high-risk drinking) and alcohol dependence’* (p46). This includes having the skills to assess the need for an intervention or to provide an appropriate referral.

Despite this guidance however, evidence from prior to the pandemic suggests a range of barriers exist in the delivery and widespread implementation of alcohol prevention work by non-specialist secondary care staff. These include time pressures, limited knowledge and awareness of AUD, and a lack of training, skills, and financial support [19–22]. Many studies also highlight that the delivery of preventative support for AUD in secondary care is hampered by wider social cultural challenges such as the stigma of heavy alcohol use and widespread belief that problematic alcohol use is a personal responsibility and represents moral failing, leading to an emphasis on individuals to manage their own care [22]. Additionally, as AUD frequently co-occurs with other physical and mental health conditions [23], non-specialist healthcare staff can find themselves ill-equipped to provide the best standard of care for these patients who have multiple and complex needs [24]. Moreover, in England, as in other health systems, the impact of COVID-19 has created additional pressures and challenges for the whole NHS, including secondary hospitals. There are more people visiting AED than before the pandemic, with longer waiting lists for treatment and fewer hospital beds [25]. There is also record dissatisfaction amongst the workforce, with more doctors now stating they want to leave the NHS than before the pandemic [26].

Given the clear need for preventive work to reduce inequalities in alcohol-related harm and the current challenges within secondary care in a post-COVID-19 context, there is value in exploring the views of secondary care staff about supporting patients with AUD since the pandemic. Moreover, the low levels of delivery of preventative support for AUD across different sites suggest there is merit in using implementation science theory [27] to support improved explanation and understanding of this situation [27, 28]. Normalisation Process Theory [29] has been used extensively in studies conducted in other health settings to understand and evaluate past and future implementation efforts e.g. [28, 30–33], including in relation to alcohol screening and brief intervention in England and Australia [30, 31]. NPT is a sociological implementation theory that identifies three domains as shaping the implementation of a new intervention or practice: contexts; mechanisms; and outcomes. Contexts refer to the *‘events in systems unfolding over time within and between settings in which implementation work is done.’* [34]; mechanisms are factors that *‘motivate and shape the work that people do when they participate in implementation processes’* [34]; outcomes refer to what changes occur when interventions are implemented. NPT is a conceptual tool and can be used at different stages of the research process [29]. In this study NPT has been used retrospectively during the analysis stage.

The aim of the present study is to use NPT to elucidate possible explanations for why the preventative practice of identifying, supporting, and referring patients with AUD to appropriate support is not consistently taking place in secondary care in the NENC in the post COVID-19 context. We also aim to make recommendations for areas that should be targeted by policy and practice initiatives.

## Methods

### Study setting

We conducted a qualitative study with health care professionals working in eight secondary care hospitals in the eight NHS Trusts in the North East and North Cumbria (NENC) region of England. The NENC experiences significant health inequalities [35], including health inequalities in alcohol-related harm. In 2021, the region had the highest reported alcohol specific and alcohol related mortality and the most alcohol related and alcohol specific admissions in England [36].

The data collection was carried out between June and October 2021. At this time, most COVID-19 restrictions had just been lifted in the NENC [37] but the impacts of COVID-19 on patients, staff and health care delivery were still ongoing.

As such, the study was planned to contribute to a baseline understanding of support for AUD in secondary care in the NENC conducted as part of a wider regional alcohol health needs assessment (2022) which would inform and direct strategic action and resource allocation in secondary care to improve alcohol-related outcomes post-COVID-19. The Principal Investigator (PI) for the study was the alcohol lead for the NENC Integrated Care System (SH), and the wider study team included representation from Primary Care, Secondary Care, Public Health, and Academia.

We used the method of qualitative semi-structured interviews to enable us to focus on issues that we wanted to explore, as well as allowing the participants flexibility to discuss the issues that were important to them [38]. We adopted a critical realist approach to the interpretation of data which purports that data can be taken as evidence for ‘real phenomena and processes’, but also recognises that the knowledge generated through qualitative research is situated and partial [39].

As part of a wider ambition to build research capacity in the study region, a novel aspect of the study design is that six junior doctors from the Gastroenterology Research and Audit through North Trainees, were trained in qualitative interview skills by a qualitative methodologist from the NIHR Applied Research Collaboration (ARC) North East and North Cumbria (NENC) and supported by members of the study team to recruit staff and carry out the interviews with secondary care clinicians.

### Participants

We used a form of stratified purposive sampling [40] as the recruitment of healthcare professionals was structured to provide insights across all the NHS Trusts in the study region, a range of clinical specialities, and a range of points across the clinical pathway, with both medical and nursing staff. As such, professionals working in AED, Medical specialties, Psychiatric Liaison (PL), Gastroenterology or Surgical specialties were eligible to participate. Junior doctor interviewers or the PI contacted potential participants either by email or face-to-face and explained the purpose of the study. People who expressed an interest were then provided with the study participant information sheet and consent form. The sampling was deemed complete when the quota of participants was met for each trust.

### Procedures

Data collection involved semi-structured interviews based on a topic guide. The topic guide was developed by the study team and was informed by the National Institute for Clinical Excellence – Quality Standard 11 [41], which contains guidance about identifying and supporting adults and young people who may have an AUD and caring for people with alcohol-related health problems (see Additional file 1).

All interviews were conducted via Microsoft Teams, lasted an average of 33 min, were audio recorded and transcribed by professional transcriptionists before being fully anonymised by KJ and IL.

### Analysis

Data analysis involved three stages:

Stage 1: Generating descriptive codes from each area of the data set

In the first stage of analysis, once all transcripts were available, in order to generate insights that could contribute to the baseline understanding of the current situation with regards to support for AUD in secondary care, one researcher (IL) used a method of thematic analysis [42] and drew on deductive and inductive reasoning to identify descriptive codes against each focus question area of the interview topic guide. This researcher read and re-read the full data set, allowing them to identify descriptive codes across staff accounts.

Stage 2: Generating descriptive and interpretive codes and themes from across the full data set

Following this, to generate insights which went beyond the question areas of the topic guide a second researcher (KJ) familiarised themselves with the data. In contrast to Stage 1, they were less restricted by the original topic guide and through a process of constant comparison began to identify both descriptive and interpretive broad thematic topic areas and codes, across the different areas of the interviews. After the first half of the interview transcripts were coded by the researcher in this way, the broad thematic topic areas were discussed with the wider study team in two meetings. In these meetings the broad topic areas and associated coding framework were refined. This refined framework was applied to future transcripts, with flexibility to add further codes as the analysis progressed. At the end of this process, a decision was made by the team to focus the interpretation for this paper on current practices around identifying, supporting, and signposting patients with AUD in secondary care hospitals because it was felt that this focus could make a meaningful contribution to the existing literature in a post-pandemic context.

Stage 3: Applying Normalisation Process Theory retrospectively to data to generate the final interpretation

To ensure the usefulness of the findings of the current analysis to support the design and delivery of future policy and practice to reduce inequalities in alcohol related harm, academic members of the team suggested using an appropriate implementation theory, namely NPT, to guide our interpretation and understanding of data from this point in the analysis [34]. NPT had not been used in the study to this point and has been used retrospectively as a sensitising, and partial structuring, device, as seen in previous comparable research e.g. [28, 43].

 [29, 34]. First, when applying NPT, we returned to the codes identified at Stage 2 to identify those that related to the practice of identifying, supporting, and signposting patients with AUD to explore how they may fit alongside the domains of NPT. At this point it was evident that most of the codes related to how implementation *contexts* and *mechanisms* were felt to adversely affect provision of support for patients with AUD. In contrast, we found negligible data related to the third NPT domain of *outcomes* (i.e. what changes occur when interventions are implemented). It was therefore agreed that applying the context and mechanisms domains could be valuable to show how contexts and mechanisms limit the implementation of the phenomena of interest. For transparency however, data not included at this stage is indicated in Additional file 2.

Next, we separated the codes generated in Stage 2 into overarching thematic areas, these were then labelled as either contexts or mechanisms. For example, poverty and austerity were labelled as contexts, and workforce skills and knowledge were labelled as mechanisms. Details of each stage of the analysis and where the codes generated at Stage 2 of the analysis were mapped, against the NPT context and mechanism domains are shown in Additional file 2.

Following this we endeavoured to align the thematic topic areas in each NPT domain into its associated constructs. It should be noted that our initial researcher-generated thematic areas aligned easily with three of the four NPT *mechanism* constructs. Conversely, as the NPT *context* constructs are a new addition to NPT theory, there were few practical examples of how these should be operationalised meaning it took more interpretive work to understand how our data mapped to these constructs. Through reflective discussions as a team, however, we identified that the researcher-generated themes aligned with three of the four context constructs. Table 1 below summarises the implementation context and mechanism constructs and identifies where our data do and do not map to these constructs. COVID-19 provides an overarching context to the study however as the timing of the interviews meant it penetrated almost all the data.

**Table 1:** Summary of the NPT implementation context and mechanism constructs and the researcher generated thematic areas and associated codes [29, 34, 44]

NPT domains and constructs	Researcher generated thematic areas and codes
Implementation Contexts - domain	
**Negotiating capacity** How contexts shape the extent to which an intervention can fit within existing ways of working.	**WIDESPREAD POVERTY AND AUSTERITY** **4.1 AUD big problem in community** **4.2 AUD common in patients** **THE PRIORITISATION OF ACUTE CONDITIONS** **8.1 Focus on acute presentations** **8.2 Resource constraints** **8.4 Time constraints**
**Strategic intentions** *How contexts affect the formulation and planning of interventions.*	**STIGMA AT A STRUCTURAL LEVEL** **6.6 Structural stigma** **9.1 No visible national commitment** **9.2 No visible NHS trust commitment** **9.3 Some national commitment** **9.4 Some NHS trusts commitment**
**Adaptive execution** *How contexts affect the way users find work arounds that make interventions possible in practice*	***No data related to this***
**Reframing organisational logic** *How existing social structural and cognitive resources shape the implementation environment.*	**INTERPERSONAL STIGMA** **3.3 Not the right time to ask about alcohol** **3.4 Patient willingness to disclose AUD** **3.6 Professional willingness to ask about AUD** **3.7 Query not the right time to ask about AUD** **6.2 Enacted – direct person to person** **6.3 Felt – interactional** **6.4 Personal responsibility (directly mentioned)** **6.5 Personal responsibility (inferred)**
**Implementation Mechanisms-domain**	
**Coherence Building** *How do people work together in everyday settings to understand and plan the activities that need to be accomplished to put an intervention and its components into practice?*	*No data related to this*
**Cognitive participation** *How do people work together to create networks of participation and communities of practice around interventions and their components?*	**WORKFORCE KNOWLEDGE AND SKILLS** **7.1 No training** **7.2 On the job training** **7.3 Some training** **7.4 Sought out or developed training**
**Collective action** *How do people work together to enact interventions and their components?*	**ROLE LEGITIMACY** **1.1 No delivery of SBI (reason not given)** **1.2 No SBI because no resource to follow up** **1.3 Partial delivery of SBI** **5.3 Lack of Awareness of services** **5.4 Little experience of signposting** **5.5 Referral to Primary care / GP** **5.6 Referral to key service in hospital** **5.7 Some awareness of services** **5.8 Some information sharing** **5.9 Someone else does signposting**
**Reflexive monitoring** *How do people work together to appraise interventions and their components?*	**PERCEIVED FUTILITY AND NEGATIVE FEEDBACK CYCLE** **2.2 Don’t know what happens to people** **2.3 Recording SBI** **2.4 Good examples of formal recording**

In keeping with the critical realist approach which recognises the situatedness of knowledge, we see researcher positionality as important to consider in the interpretation of qualitative data. Research can never be value free but, it is necessary to be explicit about where positionality might have affected the interactions [45]. The junior doctor interviewers and the PI who collected the data had experience of clinical work on the topic of the research. Indeed, the transcripts indicated that there were times when the interviewers aligned themselves or discussed their own experiences in the interviews. Some of the junior doctor interviewers recorded reflexive notes about the interviews, these were used during Stages 1 and 2 of the analysis to support interpretation, but have not been used as data. The researcher who conducted Stage 1 of the analysis has a professional background in healthcare but no direct experience of the topic area. The researcher who led the rest of the analysis has experience of carrying out research about AUD, but no clinical experience of working with people experiencing AUD. Other members of the project team have direct experience of working in hospital settings with patients experiencing AUD. Agreement amongst this heterogeneous research team about the final interpretation gives us confidence that it is grounded in the data. Moreover, this agreement amongst the research team about the final interpretation, and the congruence of findings with the existing literature on the topic of the research prior to COVID-19, gives us confidence that the insider researchers did not compromise the quality of the original empirical data.

### Findings

In total, 30 staff in the study region were interviewed across the eight NHS Trusts, including 20 nurses and 10 doctors (see Table 2) based in five departments: AED; PL; Medical; Surgical; and Gastroenterology (*n* = 6 each). Information related to participant gender and ethnicity are not available and we have not analysed the data with these as a focus. The absence of this data also helps to preserve the anonymity of participants because the geographical region of the study is named.

**Table 2:** Participant characteristics

Participant Characteristic		Number
**Job role / profession**	Nurse	20
	Doctor	10
**Department**	Gastroenterology (Alcohol lead)	6
	Medical	6
	Surgical	6
	Psychiatric Liaison (PL)	6
	Accident and Emergency Department (AED)	6
**Acute Trust Region**	Trust 1	4
	Trust 2	5
	Trust 3	3
	Trust 4	4
	Trust 5	4
	Trust 6	3
	Trust 7	4
	Trust 8	3
**Total participants**		**30**

Overall, participants’ accounts suggested that they were not consistently trying to identify AUD or assessing the need for intervention in the patients they worked with. Where any identification of AUD did take place, this appeared to often be through informal questioning rather than utilising formal, validated screening questionnaires. The following response was typical:

We’ll just ask about units a week. I know that there is a screening tool, there is a chart of some sort and it’s a physical thing that I think the alcohol and drugs nurses use on medications. So we don’t use that on a regular basis. As of now, there’s still a paper–based documenting system, but we don’t use that necessarily. (Participant 14 – Doctor, Trust 4, AED)

Conversely, some staff working in PL teams suggested they more commonly tried to identify AUD. Although again, validated screening questionnaires appeared to be used inconsistently:

Substance misuse is always an integral part of the assessment that we do. . We do have specific packs that we are trained to carry out our assessments to. I think in practice, we often don’t follow those verbatim and we will just do a free form assessment and substances are always part of that… .: “Do you consider that’s an issue for you, is it something that you want help with?” We’re always having those conversations. (Participant 8 – Nurse, Trust 2, PL)

Many staff’s accounts suggested they did not consistently signpost patients with identified AUD to a service that could provide an assessment of need or provide further care. Using NPT to frame our interpretation, in the next section we aim to highlight current practice around these phenomena and identify areas that appeared to be key barriers to implementation.

### Implementation contexts

The successful implementation of interventions requires supportive implementation environments both within and outside the settings in which they are delivered. Our data highlighted several key aspects of the implementation context/s that are barriers to the widespread implementation of asking about, supporting, and signposting patients with AUD in secondary care in the study region. As the data collection was conducted very soon after COVID-19 restrictions ended, COVID-19 was an overarching context of the staffs’ accounts.

### Widespread poverty, austerity, and the prioritisation of acute conditions – negotiating capacity

Negotiating capacity refers to how contexts shape the extent to which interventions can fit into existing ways of working [34]. Through the participants’ accounts we identified two aspects of *context* which appear to limit negotiating capacity: widespread poverty and austerity within the study region; and the focus of secondary care hospitals on the acute and presenting health needs of patients.

Most staff accounts suggested they perceived AUD to be common in the communities their hospitals covered and the patients they saw. Many staff linked the prevalence of AUD in the region to the high rates of poverty. To illustrate, Participant 23 commented that the basic provision for patients with AUD in the hospital, was in stark contrast to the apparent need in the community:

The demographic for around here, people are poor, they do drink, people do smoke,. . people take drugs a lot around here and the help, there isn’t [anything for them] it’s absolutely crazy. (Participant 23 - Nurse, Trust 6, Surgical)

While the need to support patients with AUD was perceived to have been high prior to the COVID-19 pandemic, many staff noted that they had seen a rise in patients presenting with or showing signs of AUD following the pandemic, with some suggesting that they felt that the presentations of alcohol-related morbidity and mortality were likely to increase in the future:

Our numbers [of patients with AUD] have gone up by 100% in five years. . So it’s not going anywhere, and I predict that at the beginning of next year we’re going to see huge influence on alcoholic dependence. Because we’ve already seen people who are having fits, first fits, people who were drinking prior to COVID or probably drinking too much, at high risk, not necessarily dependent and then, furloughed, have begun to drink every day and developed alcohol dependence. (Participant 25 - Nurse, Trust 7, Gastroenterology)

A small number of participants mentioned that because of the observed high levels of AUD in the study region it was harder to decide how to prioritise who to ask about alcohol. They indicated that they were unlikely to ask patients about alcohol if they were drinking at what they saw as lower levels, as they perceived most people were drinking a lot. For example, Participant 7 said:

If they were a binge drinker or they drank more than was recommended, it’s kind of like, where do you take that? How do I talk to my patients about that? Thinking about where we live, our demographic of the type of patients that we see, it’s very common that patients would drink more alcohol than the recommended. So, I guess that is the challenge of how you would approach that to the patient, without coming across like you were being judgmental or self-righteous when you’re trying to give them this advice. And actually asking them; ‘do you even see it as a problem?’ A lot of patients that you would speak to you wouldn’t even say that that is a problem. (Participant 7 - Nurse, Trust 2, Surgical)

Thus, these accounts indicated that the normalisation and prevalence of heavy drinking in some communities actively constrained the extent to which staff could integrate asking about and supporting patients with alcohol use into their day to day work .

Conversely, and illustrating how contexts can be barriers to implementation in one setting but facilitate it in others [44], some staff working in PL described how they had recently begun doing more systematic screening for AUD because it was recognised as being so prevalent in the patients they saw.

[Previously] unless alcohol was kind of front and centre and was an issue that was discussed from the get-go, it wasn’t always something that was really looked into in great detail as part of our assessments. Whereas now that we do the AUDIT, there’s an AUDIT-C tool with all patients. (Participant 4 – Nurse, Trust 1, PL)

Nonetheless, staff accounts more commonly focused on the need to tackle severe alcohol harm rather than preventative work. In-keeping with other research studies and clinical knowledge, the participants’ suggested that a key reason that patients aren’t routinely being asked about AUD in secondary care is because staff need to prioritise the presenting acute condition/s. Something which is colloquially termed ‘the rule of rescue’. Thus, any identification of AUD, where it did happen, was primarily focused on managing patients whose alcohol use was already affecting, or had the potential to affect, the treatment of their acute physical or mental illness. Participants almost always linked this to the pressurised setting and the restricted time they had to work with patients, as further limiting their capacity to address a patient’s drinking. This context is illustrated in the following quotes:

‘I’m asking [about alcohol] because it effects how I care for that patient and not necessarily about educating them’ (Participant 15 – Doctor, Trust 4, Medical).

. .I think asking about the preventative problems, and screening for problems, is something that we just don’t do. If someone comes in and they’re alcohol dependent, realistically the thing you think about most is, right well we need to make sure that we’ve got the right things for if they withdraw, you don’t think, oh well shall we see if there’s anything we can do and to be fair, you don’t really have the time, I don’t think. (Participant 6 - Doctor, Trust 2, AED)

Overall, time and the focus on acute conditions, were commonly cited by staff as key contextual factors, that limited their negotiating capacity to ask patients about alcohol and to provide follow-up support.

### Stigma at a structural level – strategic intentions

Strategic intentions refers to how contexts shape the formulation and planning of interventions. Many staff accounts suggested that they perceived there was little visible commitment to the prevention of AUD within their NHS trust or at a national NHS level. Many staff suggested they had seen no communications about providing preventative support to patients with AUD from their trust:

There’s nothing to my knowledge, Trust–wide, of how we help this cohort of patients. There doesn’t seem to be anything written in stone, on the help that we provide. (Participant 21 – Nurse, Trust 6, AED)

Others emphasised that although they had seen some communications about alcohol from their trust, these were limited. Some participants’ accounts indicated a sense of frustration that alcohol was not being prioritised by the NHS and moreover that any care offered to patients with AUD was voluntary rather than a designated part of their core work. For example, in one trust it was noted that the role of the Alcohol Lead was not formalised:

At the moment it’s almost voluntary and there’s always something else that comes along that’s more immediate, more important or seems that way. People aren’t taking the longer view that if we don’t address this problem now then the tsunami of liver disease will just continue. (Participant 10 - Doctor, Trust 3, Gastroenterology)

### Relational stigma – reframing organisational logic

Reframing organisational logic refers to the extent to which social structural and social cognitive resources shape the implementation environment [34]. The stigma which was evident at a structural level was also directly perceived to impact the care of patients with AUD at a relational level. Many staff mentioned that the identification of AUD and subsequent signposting for patients who drink heavily are obstructed because some staff perceive that heavy alcohol use is a personal failing and individual problem. Indeed, judgement or stigma was explicitly proposed by participants as one of the key reasons that AUD prevention and treatment interventions were not implemented, or attempts weren’t made to help people with AUD:

People find them incredibly frustrating and [like] they’re not real patients or people who need [help]. (Participant 4 - Nurse, Trust 4, PL)

This judgement was also seen to be compounded by austerity and the increased demands on health and social care post COVID-19, meaning those who were more challenging or difficult to help were often the easiest group to not manage.

Relational stigma appeared evident in the reluctance of some staff to speak to patients about alcohol. For example, a few participants expressed concern about how patients would respond if they were to ask them about their alcohol use because heavy alcohol consumption can sometimes be perceived by patients and wider society as a personal failing or as evidence of a lack of control:

It’s quite a personal conversation to have with somebody and you’ve got a small thin curtain between every single patient and having those conversations when everybody hears the conversation that you have in the bay, so I think that sometimes contributes to it. (Participant 24 – Nurse, Trust 7, Medicine)

Moreover, the effects of stigma seemed evident in the extent to which staff perceived people would be honest about or disclose their heavy drinking and the extent to which would subsequently make adaptions to investigate further. Some staff said that they did not have the time to build rapport with patients to generate a context where they perceived patients might be more likely to be truthful about their drinking:

It comes down to them being honest. If they say that they don’t drink a lot then we wouldn’t give any advice. (Participant 26 – Nurse, Trust 7, Surgical)

The data also suggests that the extent to which staff appeared willing to identify or support patients with AUD is related to them not seeing it as relevant to the presenting problem which relates to the prioritisation of acute conditions and the negotiating capacity.

### Implementation mechanisms

Alongside contexts, we identified a number of mechanisms that appeared to be barriers to implementation across our participants’ accounts.

### Workforce knowledge and skills – cognitive participation

All participants’ accounts suggested that there was no mandatory training within trusts to support staff to deliver alcohol prevention work. While participants acknowledged there was indeed very little mandatory training about most conditions, many staff suggested they had not been trained post-University in how to have conversations with patients about alcohol, to assess need, or how to refer and signpost on:

. . we’ve got team days where we go through mandatory training and do little courses and do all our training, but there’s nothing about alcohol on there whereas it might be quite useful because we do get a lot of patients with alcohol issues so that would be beneficial. . we’ve had no training or updates on what’s out there in the community. (Participant 9 – Nurse, Trust 2, Medical)

In a small number of trusts, some staff with a specific remit around alcohol stated they were in the process of developing training about identification within their teams and appeared optimistic about the spread and impact of this.

Where staff did ask about alcohol, a barrier to referring people with AUD to appropriate services was their limited awareness of relevant services within the community. Indeed, a few participants conveyed the sentiment of Participant 11 who described their perception of asking about alcohol in their hospital as a ‘*tick box exercise rather than purposeful tool*.’ (Nurse, Trust 3, Medical). Only a small number of participants seemed very knowledgeable about local community services; like Participant 9 above, most staff accounts suggested a lack of awareness of relevant organisations they could refer patients to. Some staff indicated that knowledge of appropriate services was made more challenging because of the frequent change in service provision and cuts and short-term commissioning of relevant voluntary and community sector services:

It is a bit vague at the moment as to exactly what they are going to do with the provider changing over. . when the Covid stuff started, they stopped coming in and just did electronic stuff. But I think they’ve started coming in again. But I don’t quite know what hours they are planning to come in, with the new changeover of people. (Participant 1 – Doctor, Trust 1, Gastroenterology)

In a context of frequent service changeovers and decommissioning, widespread poverty and austerity, and limited awareness of appropriate local services, there appeared to be a heavy reliance on referrals to primary care by staff, even when they didn’t know what primary care would offer patients. This is illustrated by this quote from Participant 15:

Sometimes if people ask me, or if I’ve found that they’ve got like deranged liver functions, I’ll often just sort of say to them, if it fits with an alcohol picture, I would say: “It does look like your alcohol use is affecting your liver, it might be something you think about cutting down,” but at that point I’m not always sure where to refer them to, so I usually end up saying you can get support from your GP. Yes. (Participant 15 – Doctor, Trust 4, Medical)

### Role legitimacy – collective action

When asked directly in the interviews about whether they felt that managing AUD was their responsibility most participants stated that it was. However, their wider accounts indicated that many participants and their colleagues relied heavily on calling on staff in other departments to manage patients with AUD who they saw as better placed to address these patients’ needs. In particular, the participants commonly suggested that alcohol nurses or other staff in gastroenterology were most able to help:

In our trust, I’m not sure if it’s the same as any others, when we do the nurse’s admission, we ask how many units they’ve had and if they score over ten then they automatically get pinged to the alcohol nurses who will come and see them. Or we refer them and call the alcohol nurses here. . (Participant 28 – Nurse, Trust 8, AED)

Staff in the site where an ACT had recently been set-up suggested that the introduction of this service had significantly improved the care that they could offer people with visible presentations of AUD and provided a clearer route for signposting. However, the reliance on this service also served to illustrate the limited support prior to this in these sites and the significant care gap at other sites who did not have this provision. Moreover, the accounts of a few participants suggested that due to the high level of need for alcohol dependent support, the ACTs appeared to have little capacity to do preventative work:

The alcohol care team nurses are building up good relationships with some of our more frequent members that are coming on ward. And then they’re able to get permission off them to do more like referrals to [community alcohol service], discussions about tapering down or alcohol reduction therapy, discussions about cognitive behavioural therapies, discussions with housing officers and things, discussions with safeguarding. . having said that, like I say they are getting an abundance of referrals daily now and I think unfortunately it’s ended up a lot bigger than they were expecting, a bit of a mammoth task. (Participant 2 – Nurse, Trust 1, Medical)

In contrast to staff in other departments, as mentioned above, staff from PL teams suggested that identifying patients’ patterns of alcohol use, usually through formalised screening, had relatively recently become part of their core work. Nonetheless, the focus was still on management of AUD rather than prevention, as most indicated that the implementation of this was in response to the prevalence of heavy drinking in the patients they saw. Here the mechanism of collective action appears to be shaped by the context of poverty and austerity.

### Perceived futility and negative feedback cycle – reflexive monitoring

Participants’ accounts indicated that they had little information about the outcomes of the people that they saw with AUD. Some staff mentioned that the only time they saw patients again, whether or not they delivered an intervention, was when they re-attended. The following response was typical:

We put them on file with the GP letter, and we don’t know what happens after that. (Participant 26 – Nurse, Trust 7, Surgical)

In the context of this perceived futility, staff appeared to find it difficult to have hope for patients when they experienced only negative reinforcement. Compounding this it was also evident that the recording of information about alcohol use and any advice or signposting were limited in most departments. Although some PL services and some trusts seemed to be trying to record screening more systematically at the time of the research, it was still not mandatory and was not always prioritised as the following quote illustrates:

[We] have the AUDIT -C put on e-records, and that provided some challenges as well. . there’s a lot of things that are recorded, you get a lot of alerts, we know that. . staff just tap off them, if they’re not mandatory, So, it was about trying to sell it is an important message. (Participant 25 - Nurse, Trust 7, Gastroenterology)

Here again we see the link between contexts and mechanisms whereby the lack of systematic recording of patients’ alcohol use is likely to be influenced by the context of structural stigma and its impact on strategic intentions.

## Discussion

This paper reports the findings of a collaborative study between practitioners, policy makers, and academics which aimed to explore the challenges to the delivery of identification, support, and subsequent signposting for AUD in the secondary care settings in the NENC region post- COVID-19. Our findings broadly concur with what was already known about the challenges of implementing identification and support for AUD in secondary care hospitals prior to the COVID-19 pandemic. For example, the persistent contextual challenge of time pressures, and the lack of key enabling mechanisms, such as having a workforce with the skills and knowledge to confidently ask about alcohol and signpost patients appropriately [22]. However, our findings extend existing evidence by highlighting some additional barriers to alcohol prevention work in secondary care in the post-COVID-19 context. Moreover, the use of theory, specifically NPT domains, enables us to illuminate the interplay of context and mechanisms which make implementation of AUD care especially difficult in this setting.

A key contribution of this study to the extant literature is that it provides empirical evidence of how COVID-19 has served to amplify the challenges already experienced by secondary care staff trying to delivery preventative alcohol work in hospital settings. Many staff indicated that the sheer scale of people presenting with possible AUD since COVID-19, meant they did not have the time to ask people or to prioritise asking people about alcohol. Where people were identified as experiencing AUD, provision of effective signposting and support for patients was adversely affected by lack of staff awareness about relevant care providers and lack of capacity in local services due to the impact of austerity and cuts to public services. Two trusts in the study region had ACTs in place at the time of the interviews, as part of the wider NHS commitment to reduction alcohol harm in England [16]. This appeared to have increased the capacity of the non-specialist workforce at these two sites to refer patients identified as experiencing AUD onto appropriate specialist support. However, a tentative, but notable, finding of this study was that while ACTs were making a difference in these trusts for those with existing alcohol dependence, they were limited in their capacity to deliver more preventative work around AUD (initially part of their remit) due to the high level of need amongst the dependent patient population. This warrants further exploration, with further insights potentially to come via the wider programme of work around ACTs that is currently ongoing in England [46]. Overall, the study provides empirical evidence that the implementation of the preventative practices to support a reduction in AUD may be particularly difficult in areas of deprivation such as the NENC meaning that inequalities are likely to be widening with other more affluent regions.

Stigma, the process of marking certain groups as being somehow contagious or of less value than others [47], is internationally recognised as a significant constraining factor to the delivery of compassionate and appropriate healthcare for patients with AUD and other substance use in secondary care and other health and social care settings [47, 48]. In this study we chose to approach stigma as a structural and relational concept, seeing relational stigma as developing from structural stigma [49]. The role of structural stigma for limiting the implementation of identifying, supporting, and signposting patients with AUD was striking, as our data highlighted that the prevention of heavy alcohol use does not appear to be a visible priority within individual trusts, and arguably the wider NHS. Limited resources were perceived available for this area of care, and little visible commitment to support patients with AUD despite the scale of the problem. Stigma was also evident at a relational level in our participants accounts of the interactions between staff and patients, notably staff’s reluctance to ask about alcohol use and their perception that patients did not want to disclose their AUD. However, it should be noted that many of the staff who took part in the study suggested that they did not perceive patients in this way yet continued to struggle to provide alcohol prevention care. Thus, this relational stigma is likely an important, but only partial explanation for limited care provision. Nonetheless, our findings suggest that structural stigma is one of the main barriers to the identification of alcohol use and care in secondary care settings in the NENC. This echoes the damning findings of the ‘Remeasuring the Units’ report, also published since the pandemic, that argued that stigma contributes to the missed opportunities in secondary care for patients who ultimately die from alcohol-related liver disease [5].

This study was conducted primarily as a vehicle to understand and bring about change in workforce practice around the prevention of alcohol harm in NENC secondary care services. It was an integral component of a broader Health Care Needs Assessment (2022) on alcohol undertaken in response to increasing levels of alcohol harm in this region of the UK, which led to recommendations over four overarching themes: service delivery; workforce; data; and leadership from the healthcare system. The results of the study have directly shaped the regional strategy for the reduction of alcohol harm, a key element of which is the integrated alcohol workforce strategy for the NENC which aims to better support the NHS workforce to prevent alcohol harm through: increased awareness of the Chief Medical Officer alcohol guidance; improved pathways to community-based alcohol treatment and recovery support; workforce training and development; and support for staff to address their own drinking. The evidence highlighting the importance of stigma have additionally led to a strategic drive for senior leaders to acknowledge the impact alcohol has on their organisation and the communities they serve, and to take action to work in partnership to reduce this. There is also cross-system support to tackle relational stigma, initially though a co-ordinated multi-agency media campaign.

Overall, our interpretation has signalled areas of policy and practice which can be targeted to try to increase the uptake of these preventive strategies in the secondary care settings. However, ultimately the findings illustrate that the challenge for implementation of these evidence based preventative measures is not just upskilling the workforce or increasing resources. It also indicates that we need to address the complex interplay of contextual factors and implementation mechanisms which have been compounded by the pandemic and contribute to reinforcing and increasing existing inequalities. The works contributes to calls for a multi-layered response to reducing alcohol harm and wider cultural change for how alcohol use and substance use is perceived.

### Study strengths and limitations

A strength of the study is that it was undertaken in an area experiencing some of the greatest inequalities from the COVID-19 pandemic. This allowed us to see the challenges to delivering preventative work in these contexts, which might be similar in other regions. A further strength is that mapping the empirical data onto an evidence-based implementation theory, which has been widely use in different settings, enabled us to focus on the aspects of the implementation, that are likely to be important across other settings too. Framing the interpretation using the NPT domains has helped us to emphasise how contexts and mechanisms interact to make the implementation at this particular time and place difficult. A key limitation of the study is that as it was based in one region of England, we cannot know for sure if these insights are transferrable beyond this context.

## Conclusions

Secondary care hospitals are an important setting for the delivery of preventative care for AUD, due to the frequency with which AUD co-occurs with other physical and mental health conditions. Prior to the pandemic there was evidence that non-specialist healthcare staff can find caring for patients with alcohol-related presentations difficult, meaning that identifying, supporting, and that signposting patients was happening inconsistently. In this study, we highlight the additional challenges facing secondary care staff due to post-pandemic pressures and the significant rise in alcohol-related harm in some regions such as the NENC. Thus, whilst the mechanisms for implementing alcohol prevention work in secondary care need attention, our findings suggest that the greatest barrier is contextual, including widespread structural stigma.

### Electronic supplementary material

Below is the link to the electronic supplementary material.

Supplementary Material 1

Supplementary Material 2

## Data Availability

No datasets were generated or analysed during the current study.

## Abbreviations

NPT: Normalisation Process Theory
ACT: Alcohol Care Teams
NENC: North East and North Cumbria
AUD: Alcohol Use Disorder
AED: Accident and Emergency Department
PL: Psychiatric Liaison Teams
AUDIT: Alcohol Use Disorders Identification Test
AUDIT-C: Alcohol Use Disorders Identification Test Consumption
