# Supplementary material for: Understanding the challenges of identifying, supporting, and signposting patients with alcohol use disorder in secondary care hospitals, post COVID-19: a qualitative analysis from the North East and North Cumbria, England
Source: BMC Health Serv Res. 2024 Jul 1;24:772. doi: 10.1186/s12913-024-11232-4 (PMC11218181; doi:10.1186/s12913-024-11232-4)
Supplement: Supplementary file 1 — Supplementary Material 1 [file 12913_2024_11232_MOESM1_ESM.docx]

**Provision of alcohol care in Acute Hosptial Trusts**

**Topic guide: Frontline clinicians**

**Introduction**

- Thank you for taking the time to meet and share your experiences and views about how care is delivered for patients at risk of alcohol harm in your Trust
- Anything you share here today will be anonymised.
- Can I check that you have read the study participant information sheet, do you have any questions for me?
- Work through written consent.
- There are no right or wrong answers the interview is about your views and experiences.
- Are you ok for me to turn the recorder on and start the interview?

**[A] Alcohol Awareness Training**

*First of all we are interested in the alcohol awareness training available to staff in your Trust:*

1. Please could you tell me about the alcohol awareness training you have had while working in this trust? *(Probe: length, who delivered it, was it mandatory?)*
2. To what extent did this training change your views or opinions about managing alcohol in your role?
3. Overall, what do you think staff attitudes are like to people with problem drinking in the Trust?

**[B] Conversations with patients about alcohol**

*We are interested in whether and how staff have conversations with patients about their drinking:*

1. Please can you tell me about times when you ask people about their drinking, or give them advice about drinking, as part of your job? *(Probe: How does that happen? What advice to they give, screening? Interventions? What are the sources of information for your advice)*
2. Could you tell me about any training you have had about how to carry out alcohol screening and brief interventions? *(Probe: when, by who, was this mandatory?)*
3. To what extent do you see it as your responsibility to ask patients about alcohol?

**[C] Referrals to other services**

*We are interested in referral pathways for heavy alcohol use:*

1. In your role, who would you refer patients to if they tell you about their problem drinking?
2. Please could you tell me about any pathways or resources you are aware of in the Trust to help with this?
3. What happens with people who have mental health problems as well as problem drinking?
4. How would you know how to help a child or young person, or a pregnant woman with problem drinking?

**[D] Conversations with families / carers about alcohol**

*We are also interested in the information and support offered to families and carers of people who misuse alcohol:*

1. Do you have any ways to identify carers of patients with problem drinking?
2. What information would you usually give to these carers?
3. How does the Trust work with children and carers of people with problem drinking to reduce their risk of harm?

**[E] Medical Assisted Alcohol Withdrawal**

*We want to find out about treatment offered for people needing medically assisted alcohol withdrawal:*

1. Are you aware of any special beds or staff to care for people in withdrawal in the Trust?
2. Are you aware of protocols and / or training for managing people in withdrawal? *(e.g. to guide on prescribing and observations)*
3. Do you have any concerns about the way withdrawal is managed in the Trust at the moment?
4. Where would you say withdrawal is best managed?

**[F] Trust policy about alcohol related harm**

*Now we’d like to find out a bit about what your Trust has in place to support all of the aspects of alcohol related harm we have asked about:*

1. Are you aware of whether the Trust has an alcohol plan and a policy? (probe: how effective do you think these are at helping manage people’s needs)
2. How would you know what to do if you were worried about your own or a colleague’s drinking? (probe: awareness of policy on staff drinking)
3. Who is the lead for alcohol care in the Trust? What is their job role? Do you think they help to guide the Trust’s management of people with problem drinking?

**[G] Joint working / Partnership arrangements**

*Finally we’d like to find out about any joint working/partnership arrangements with other organisations that support people at risk of alcohol related harm:*

1. Are you aware of any work the Trust does with other organisations like the mental health trust and the local authority or community providers around alcohol?
2. Do you think the Trust’s care of people with problem drinking is ‘joined up’?
3. What would you see as you your Trusts role in the wider system to prevent alcohol harm?

# **Closing question**

1. Is there anything else you want to say about the topic of this interview, or about the things we have discussed?

# **End of interview**

Thank participant for their time and switch off recorder.
