# Supplementary material for: Understanding the challenges of identifying, supporting, and signposting patients with alcohol use disorder in secondary care hospitals, post COVID-19: a qualitative analysis from the North East and North Cumbria, England
Source: BMC Health Serv Res. 2024 Jul 1;24:772. doi: 10.1186/s12913-024-11232-4 (PMC11218181; doi:10.1186/s12913-024-11232-4)
Supplement: Supplementary file 2 — Supplementary Material 2 [file 12913_2024_11232_MOESM2_ESM.docx]

Acronyms used:

AUD - Alcohol Use Disorder

NHS - National Health Service

CQUIN - Commissioning for Quality and Innovation

SBI - Screening and Brief Intervention

GP - General Practitioner

Stage 1: A table of the descriptive codes generated for each area of the topic guide

| **Question area of topic guide** | **Codes** |
| --- | --- |
| 1. Alcohol Awareness Training |  |
|  | A1. Change your views |
|  | A2. Staff attitudes |
| 1. Conversations with patients about alcohol |  |
|  | B1. Brief intervention training |
|  | B2. Responsibility to ask patients about alcohol |
|  | B3. Ask people about their drinking |
| 1. Referrals to other services |  |
|  | C1. Refer patients |
|  | C2. Pathways or resources |
|  | C3. Mental health problems or problem drinking |
|  | C4. Child, young person, pregnant woman |
| 1. Conversations with families or carers about alcohol |  |
|  | D1. Identify carers of patients |
|  | D2. Information to carers |
|  | D3. Work with children and carers |
| 1. Medically Assisted Withdrawal |  |
|  | E1. Special beds or staff for withdrawal |
|  | E2. Protocols or training for managing withdrawal |
|  | E3. Withdrawal is best managed |
|  | E4. Any concerns withdrawal |
| 1. Trust policy about alcohol related harm |  |
|  | F1. Alcohol plan and policy |
|  | F2. Worried about your own or colleagues drinking |
|  | F3. Lead for alcohol care |
| 1. Joint working or partnership arrangements |  |
|  | G1. Work with other organisations |
|  | G2. Care is joined up |
|  | G3. Role in wider system |

Stage 2: A table to show the broad topic areas and codes generated from the full data set prior to the application and engagement with NPT.

| **Broad topic area** | **Code^[[1]](#footnote-1)^** |
| --- | --- |
| 1.Delivery of SBI |  |
|  | 1.1 No delivery of SBI (reason not given) |
|  | 1.2 No SBI because no resource to follow up |
|  | 1.3 Partial delivery of SBI |
| 2.Formal Recording of alcohol related harm |  |
|  | *2.1 CQUIN targets* |
|  | 2.2 Don’t know what happens to people |
|  | 2.3 Recording SBI |
|  | 2.4 Good examples of formal recording |
| 3.Relational Challenges |  |
|  | *3.1 Engaging families* |
|  | *3.2 Judgemental approach to SBI* |
|  | 3.3 Not the right time to ask about alcohol |
|  | 3.4 Patient willingness to disclose AUD |
|  | *3.5 Personal or own experience of AUD* |
|  | 3.6 Professional willingness to ask about AUD |
|  | 3.7 Query not the right time to ask about AUD |
|  | *3.8 Skewed perception of an alcohol problem* |
| 4.Scope of AUD |  |
|  | 4.1 AUD big problem in community |
|  | 4.2 AUD common in patients |
|  | *4.3 ‘Basic at best’ – examples of poor care* |
|  | *4.4 Recent improvement in service provision for AUD* |
|  | 4.5 Increase in AUD since COVID-19 |
|  | *4.6 Reduction in service provision for AUD* |
|  | 4.7 Should be doing more for patients with AUD |
| 5.Signposting and Referral to treatment |  |
|  | *5.1 Concern about Community Alcohol Service* |
|  | *5.2 Digitised Referral Pathway* |
|  | 5.3 Lack of Awareness of services |
|  | 5.4 Little experience of signposting |
|  | 5.5 Referral to Primary care / GP |
|  | 5.6 Referral to key service in hospital |
|  | 5.7 Some awareness of services |
|  | 5.8 Some information sharing |
|  | 5.9 Someone else does signposting |
| 6.Stigma |  |
|  | *6.1 Developing empathy* |
|  | 6.2 Enacted – direct person to person |
|  | 6.3 Felt – interactional |
|  | 6.4 Personal responsibility (directly mentioned) |
|  | 6.5 Personal responsibility (inferred) |
|  | 6.6 Structural stigma |
| 7.Training |  |
|  | 7.1 No training |
|  | 7.2 On the job training |
|  | 7.3 Some training |
|  | 7.4 Sought out or developed training |
| 8.Treatment not Prevention |  |
|  | 8.1 Focus on acute presentations |
|  | 8.2 Resource constraints |
|  | *8.3 See value in more prevention work* |
|  | 8.4 Time constraints |
| 9.Visibility of commitment to AUD |  |
|  | 9.1 No visible national commitment |
|  | 9.2 No visible NHS trust commitment |
|  | 9.3 Some national commitment |
|  | 9.4 Some NHS trusts commitment |
|  | *9.5 Visibility – comparison with smoking* |

Stage 3: A table to show how the codes from Stage 2 of the analysis were combined to generate broad thematic areas to form our interpretation presented in the manuscript, and how these thematic areas were then mapped to the NPT implementation contexts and mechanisms domains

The following codes from Stage 2 are used in the introduction to the Findings section 1.3, 4.5, 4.7,

| **Broad thematic area** | **Code (number from Stage 2)** |
| --- | --- |
| **IMPLEMENTATION CONTEXTS (NPT – domain)** | |
| Widespread poverty and austerity |  |
|  | 4.1 AUD big problem in community |
|  | 4.2 AUD common in patients |
| The prioritisation of acute conditions |  |
|  | 8.1 Focus on acute presentations |
|  | 8.2 Resource constraints |
|  | 8.4 Time constraints |
| Stigma at a structural level |  |
|  | 6.6 Structural stigma |
|  | 9.1 No visible national commitment |
|  | 9.2 No visible NHS trust commitment |
|  | 9.3 Some national commitment |
|  | 9.4 Some NHS trusts commitment |
| Interpersonal Stigma |  |
|  | 3.3 Not the right time to ask about alcohol |
|  | 3.4 Patient willingness to disclose AUD |
|  | 3.6 Professional willingness to ask about AUD |
|  | 3.7 Query not the right time to ask about AUD |
|  | 6.2 Enacted – direct person to person |
|  | 6.3 Felt – interactional |
|  | 6.4 Personal responsibility (directly mentioned) |
|  | 6.5 Personal responsibility (inferred) |
| **IMPLEMENTATION MECHANISMS (NPT- domain)** | |
| Workforce knowledge and skills |  |
|  | 7.1 No training |
|  | 7.2 On the job training |
|  | 7.3 Some training |
|  | 7.4 Sought out or developed training |
| Role legitimacy |  |
|  | 1.1 No delivery of SBI (reason not given) |
|  | 1.2 No SBI because no resource to follow up |
|  | 1.3 Partial delivery of SBI |
|  | 5.3 Lack of Awareness of services |
|  | 5.4 Little experience of signposting |
|  | 5.5 Referral to Primary care / GP |
|  | 5.6 Referral to key service in hospital |
|  | 5.7 Some awareness of services |
|  | 5.8 Some information sharing |
|  | 5.9 Someone else does signposting |
| Perceived futility and negative feedback cycle |  |
|  | 2.2 Don’t know what happens to people |
|  | 2.3 Recording SBI |
|  | 2.4 Good examples of formal recording |

1. Note that the codes not included in the final interpretation once NPT was applied (at Stage 3) are highlighted in italics [↑](#footnote-ref-1)
